# Artificial intelligence techniques applied to anxiety disorders recognition: a systematic review

**DOI:** 10.3389/fdgth.2025.1646724

**Published:** 2025-11-24

**Authors:** Edgar Degante-Aguilar, Roberto Angel Melendez-Armenta, Giovanni Luna-Chontal, Francisco Javier Fernandez-Dominguez

**Affiliations:** Affective Computing and Educational Innovation Laboratory, Division of Graduate Studies and Research, Tecnológico Nacional de México-Instituto Tecnológico Superior de Misantla, Misantla, Veracruz, Mexico

**Keywords:** anxiety disorders, artificial intelligence, PICO, PRISMA-statement, mental health

## Abstract

**Objective:**

This Systematic Review aims to provide a comprehensive analysis of the current state of anxiety disorder detection methods using Artificial Intelligence (AI), focusing on their accuracy and the scope of research. This review is tailored for researchers, clinicians, and technology developers seeking to understand the advancements in AI-driven mental health diagnostics.

**Methodology:**

A Systematic Review was conducted following the PRISMA Statement guidelines, utilizing databases such as IEEE Xplore, PubMed, ScienceDirect, and SpringerLink. The review included studies focusing on the diagnosis of anxiety disorders using quantitative data and AI techniques, excluding those solely focused on depression or lacking experimental datasets.

**Results:**

A total of 119 studies were analyzed, revealing the application of Machine Learning and Deep Learning techniques in detecting anxiety disorders from diverse data sources, including self-reports, physiological data, and social network data. The findings indicate that AI-driven methods demonstrate higher accuracy compared to traditional anxiety disorder detection tests, providing valuable insights for clinicians and researchers exploring improved diagnostic tools.

**Conclusions:**

This review highlights the critical role of AI in optimizing the detection and treatment of anxiety disorders. It offers a current and detailed overview of advancements in this field, making it a key resource for researchers, healthcare professionals, and technology developers aiming to integrate AI into mental health practices. The synthesis of findings provides a clear understanding of the current landscape and potential future directions in AI-based anxiety detection.

**Systematic Review Registration:**

https://www.crd.york.ac.uk/PROSPERO/view/CRD420251026205, identifier CRD420251026205.

## 1 Introduction

Mental health is a state of well-being for every human being and essential for full development in all areas of life. The WHO indicates that it influences our actions and functions as an undeniable condition for every human being, as important as our physical health; essential for relating positively, contributing, building, obtaining a sense of satisfaction, and empathizing with people. However, in its absence, the individual may present obvious negative psychological and physical symptoms and even the appearance of serious medical conditions such as post-traumatic stress disorder, sleep disorders, cardiovascular diseases, and anxiety disorders (1). The WHO (2022) relates the state of mental health to skills, habits, and emotions generated by the exchange of experiences with other people, so if you have poor mental health, it may be the product of the influence of psychological and physical factors.

There are various internal and external factors that disturb its balance and give rise to a series of disorders that negatively impact the well-being of people. One of the most common and prevalent disorders today is anxiety, which has become a global public health problem, significantly affecting the quality of life of those who suffer from it WHO. Its main characteristic consists of a disproportionate alert response to situations perceived as threatening, generating a variety of psychological and physical symptoms, such as excessive worry, irritability, restlessness, and agitation (2). Early and accurate identification of anxiety is crucial to initiate timely therapeutic interventions and prevent long-term complications, such as cardiovascular diseases and even suicide (2).

The research results demonstrate a broad panorama of diverse studies carried out worldwide. For example, (3) identified a considerable increase in medical incidents related to mental health, in addition to finding 46 studies for depressive disorders and 29 related to anxiety disorders globally. These results led to the work of other researchers in the area focusing on anxiety disorders. In this sense, (4), with reference to (5), explains that there is a correlation between anxiety disorders and the emotional intelligence of the individual; and underlines a higher level of anxiety in women because they express, to a greater extent, their emotions regarding situations in their context. On the other hand, anxiety is considered a state of alert caused by situations that generate fear and excessive worry at any time in life, and they are of a psychological nature (2). This defensive response, although useful in some cases, can cause a series of uncomfortable emotions: restlessness, irritability, hypervigilance, agitation, worry, among others. The findings of (3) recorded a significant increase in cases of depression and anxiety. These disorders, especially anxiety, can manifest themselves in various ways, from mild restlessness to a state of constant alertness, as described by (2).

In the Mexican context, all federal entities have their own Health Law, however, (6) reports on 14 federal entities in the national territory that have a Mental Health Law, representing 43.8%, and only five states define mental health within their Health Laws. Veracruz stands out for comparing the term mental health with the optimal state of complete mental, social, and emotional well-being. The actions to implement strengthening services in Mental Health for citizens correspond to the functions of the National Mental Health Council that has been operating since 2004. This body helps coordinate treatment policies. However, the unique context of everyone, such as the scarcity of economic resources, the level of education, even the stereotypes of society, among other factors, add complexity to receive adequate care. The selected studies show that, in Mexico, through the application of tests to identify anxiety disorders, (7) they found a higher percentage of people with 58.89% in the Beck Depression and Anxiety Inventory test and 37.5% with the BAI test (8). 11.10% of men in contrast to 25.3% of women in the same study were classified with severe anxiety during the confinement stage caused by COVID-19, so the methods used were aligned with the health regulations and must currently be applied again to evaluate in the post-covid stage.

The application of any anxiety diagnosis generates biases in the quality of information due to the subjectivity of symptoms and comorbidity with other mental health disorders. Consequently, traditional diagnostic tools, such as clinical interviews and questionnaires, can be limited by interviewer bias and the lack of objectivity in the responses provided by the subjects involved. Therefore, technological tools, mostly software, represent an innovation in the detection of mental health disorders (9) since they allow the analysis of large amounts of data from various sources, such as self-reports (10), physiological data, interaction on social networks, among others, to identify patterns and characteristics associated with anxiety. The main strength lies in the possibility of developing more objective, accurate, and accessible detection tools that could be implemented in clinical and community settings.

Therefore, this systematic review seeks to examine research trends related to the detection of anxiety disorders by analyzing the AI algorithms used, their accuracy, and future research. In such a way that, by understanding their potential and limitations in this field, key areas for future research and development of tools that improve the identification and treatment of anxiety are focused, thus contributing to the well-being of people affected by anxiety disorders.

## 2 Materials and methods

This systematic review was performed according to the PRISMA statement.

Table 1 shows the Thesaurus defined for the development of the Systematic Review. The selection of terms and synonyms was carried out through a process of analyzing the definitions and their context of application.

**Table 1:** Thesaurus for the development systematic review.

Term	Synonyms and related terms
Artificial intelligence	Machine learning, deep learning, redes neuronales, natural language processing (NLP), computer vision (CV)
Anxiety disorders	Generalized anxiety disorder (GAD), panic disorder, phobias, social anxiety disorder, obsessive-compulsive disorder (OCD), post-traumatic stress disorder (PTSD)
Recognition	Detection, diagnosis, classification, prediction, evaluation
Data	Physiological data (e.g., heart rate, galvanic skin response), neuroimaging data (e.g., fMRI, EEG), voice data, text data (e.g., social media, questionnaires), behavioral data
AI methods	Support vector machines (SVM), decision trees, random forests, convolutional neural networks (CNN), recurrent neural networks (RNN), transformers, sentiment analysis
Ethic	Data privacy, algorithmic bias, informed consent

### 2.1 Research question

The growing prevalence of anxiety disorders in the global population has driven the search for innovative technological solutions to improve their early detection and diagnosis. In this context, tools have emerged in recent years to analyze large volumes of data and detect complex patterns that may be indicative of psychological disorders (11). However, the diversity of available AI techniques and the methodologies used in their development raises questions about their effectiveness and applicability in different contexts. Therefore, the review carried out focuses on two fundamental questions: RQ1, which seeks to identify which AI techniques have shown better performance in the identification of anxiety disorders in diverse populations; and RQ2, which explores the methodologies and approaches used to train and validate these techniques. Addressing these questions will highlight best practices and possible areas for improvement in the recognition of anxiety disorders.

RQ1. What Artificial Intelligence techniques show the best performance in identifying anxiety disorders in diverse populations? RQ2. What methodologies and approaches have been adopted to train and validate Artificial Intelligence techniques in identifying anxiety disorders?

Table 2 presents in detail the application of the PICO model (12) to both research questions, providing a clear description of the key elements for each. This approach not only facilitates the structuring of the literature search and selection process but also ensures that the answers obtained are specific, relevant, and aligned with the objectives set out in the systematic review.

**Table 2:** PICO elements of research questions RQ01 and RQ02.

PICO element	RQ1: AI techniques with better performance	RQ2: Methodologies and approaches for training and validation
P	Individuals in diverse populations with a clinical diagnosis or suspicion of anxiety disorders.	Data related to individuals with anxiety disorders used in training and validation studies.
I	Application of AI techniques	Methods and strategies for training and validating models.
C	Comparison between different techniques used in the studies	Comparison of the different methodologies and approaches used.
O	Identification of performance based on metrics such as accuracy, sensitivity, specificity, F1-score.	Description of the most used methodologies and approaches.

## 3 Methodology

The systematic review is based on the PRISMA Statement checklist and included studies published in the last five years (from January 2019 to September 2024) related to the identification of anxiety disorders using Artificial Intelligence techniques in scientific articles hosted on databases IEEE Xplore, ScienceDirect, PubMed, and SpringerLink, respectively. To obtain the publications, advanced search equations were used, taking care not to exceed the use of two AND operators to ensure greater precision. The following search terms were used in these equations: anxiety, Machine Learning, Artificial Intelligence, and Deep Learning. In this way, comprehensive coverage of the various methodologies and technological approaches applied in the identification of anxiety disorders was achieved. This selection of keywords ensures the inclusion of relevant studies that use advanced Artificial Intelligence techniques to address anxiety disorder problems.

### 3.1 Search strategy

The choice of search terms and date range was based on relevance to the detection of anxiety using AI techniques and the need to include recent research with a high impact on the field of study. The search strategy was developed by Edgar Degante-Aguilar and Giovanni Luna-Chontal. Additionally, articles that do not address anxiety and that focus solely on depression or lack information generated in the experiments were excluded. Any disagreements in the article selection were resolved through discussion and consensus between the two authors. In case of persistent disagreement, a third external arbitrator, an expert in the research area, was designated to make the final decision. Table 3 shows the search strategies in detail, indicating the type of study, database/search engine, and the specification of the terms. Table 3 presents the fundamental search parameters to guarantee that the process is rigorous, reproducible, and exhaustive. They are defined based on the research objective and serve to delimit the scope of the search, identifying the most relevant sources and reducing the risk of bias. Their proper selection and description ensure that the results obtained are representative and relevant to answer the research questions posed.

**Table 3:** Fundamental search parameters.

Parameter	Specification
Search period	January 2019 to September 2024
Databases/Academic search engines	Scopus, ScienceDirect, PubMed, IEEE Xplore, SpringerLink
Keywords	Artificial intelligence (AI)
Search terms	TITTLE-ABS-KEY/ALL FIELDS/PUBYEAR AFT
Search methods	Boolean operators (AND, OR)
Type of studies	Original, scientific articles
Discipline	Computer science, artificial intelligence
Subdiscipline	No restriction
Language	English and Spanish
Country	No restriction
Review	Peer review
Select of studies	Conducted by Edgar Degante Aguilar and Giovanni Luna Chontal, verified by Roberto Ángel Meléndez-Armenta, refereed by Francisco Javier Fernández-Domínguez.

Table 4 displays the results of an exhaustive search conducted in the PubMed bibliographic database. It focused on identifying the number of scientific articles that explore the intersection between three key areas: artificial intelligence, anxiety disorders, and tasks related to medical information processing. The results are broken down into six sub-equations, each representing a specific combination of search terms using MeSH controlled terminology. The first sub-equation counts the total number of AI-related articles, the second focuses on anxiety disorders, and the third covers a broad set of terms related to tasks such as recognition, detection, diagnosis, classification, prediction, and evaluation. The following 4, 5, and 6 combine the previous terms, with the last two restricting the search to a specific period (2019–2024) and, in the case of Equation 6, filtering the results to include only open access articles. The numbers presented in the results column indicate the number of articles that meet the criteria of each sub-equation, offering a quantitative view of the frequency with which they have been worked on by other researchers.

**Table 4:** Search results from advanced search equations per database.

Database	Number	Subequation	Results
IEEE Xplore	1	(“Artificial intelligence” OR “machine learning” OR “deep learning” OR “neural networks” OR “natural language processing” OR “computer vision”)	807,411
	2	(“Anxiety disorders” OR “generalized anxiety disorder” OR “panic disorder” OR phobias OR “social anxiety disorder” OR “obsessive-compulsive disorder” OR “post-traumatic stress disorder”)	2,303
	3	(recognition OR detection OR diagnosis OR classification OR prediction OR evaluation)	1,000,000+
	4	#1 AND #2 AND #3	641
	5	#1 AND #2 AND #3 (last five years: 2019 to 2024)	585
	6	#1 AND #2 AND #3 (last five years: 2019 to 2024, journals and early access articles)	93
PubMed	1	(“Artificial intelligence”[MeSH] OR “machine learning”[MeSH] OR “deep learning”[MeSH] OR “neural networks (computer)”[MeSH] OR “natural language processing”[MeSH] OR “computer vision”[MeSH])	209,505
	2	(“Anxiety disorders”[MeSH] OR “generalized anxiety disorder”[MeSH] OR “panic disorder”[MeSH] OR phobias[MeSH] OR “social anxiety disorder”[MeSH] OR “obsessive-compulsive disorder”[MeSH] OR “stress disorders, post-traumatic”[MeSH])	135,671
	3	(recognition[Title/Abstract] OR detection[Title/Abstract] OR diagnosis[Title/Abstract] OR classification[Title/Abstract] OR prediction[Title/Abstract] OR evaluation[Title/Abstract])	5,281,884
	4	#1 AND #2 AND #3	149
	5	#1 AND #2 AND #3 AND (“2019/01/01”[Date - Publication] : “2024/09/28”[Date – Publication])	149
	6	#1 AND #2 AND #3 AND (“2019/01/01”[Date - Publication] : “2024/09/28”[Date – Publication]) With filter, Free full text	91
ScienceDirect	1	(Artificial intelligence OR machine learning OR deep learning)	184,477
	2	(Anxiety disorders OR generalized anxiety disorder)	38,065
	3	(Recognition OR detection OR classification OR prediction)	1,000,000+
	4	#1 AND #2 AND #3	120
	5	1 AND 2 AND 3 (last five years: 2019 to 2024)	
SpringerLink	1	(“Artificial intelligence” OR “machine learning”)	10,000+
	2	(“Anxiety disorders”)	10,000+
	3	(Title:(“prediction” OR “classification”))	10,000+
	4	(“Artificial intelligence” OR “machine learning”) AND (“anxiety disorders”) AND (Title:(“prediction” OR “classification”))	154
	5	#1 AND #2 AND #3 (last five years: 2019 to 2024)	112
	6	#1 AND #2 AND #3 (last five years: 2019 to 2024, type: Article)	84

Likewise, this table presents the results of a search carried out in the ScienceDirect database. This search focused on identifying the number of scientific articles that address the intersection between three main areas: artificial intelligence, anxiety disorders, and tasks related to recognition, detection, classification, or prediction. The results are broken down into five parts, each representing a specific combination of search terms. The first counts the total number of AI-related articles, while the second focuses on anxiety disorders. The third covers a broad set of terms related to data processing tasks. Subequations 4 and 5 combine the previous terms, with the latter restricting the search to the last five years. The numerical results indicate the number of articles found for each subequation, this showing the frequency with which these topics are combined in scientific research.

In the same table presents the results of a search carried out in the SpringerLink database. The results are broken down into six parts, each representing a specific combination of search terms. The first three sub-equations count the total number of articles related to AI, anxiety disorders, and prediction or classification tasks, respectively. The remaining sub-equations function as a filter to specify and narrow down the results. This provides a general idea of the amount of research existing in each of the areas studied.

Additionally, presents the results of a comprehensive search performed in the IEEE Xplore database. The results are broken down into six sub-equations, each representing a specific combination of search terms. The first three sub-equations count the total number of articles related to AI, anxiety disorders, and tasks of recognition, detection, diagnosis, classification, prediction, or evaluation, respectively. The last two sub-equations further refine the search, limiting it to the last five years (2019–2024) and, in the case of sub-equation 6, specifying that the results must correspond to articles published in journals or in early access.

The search terms were carefully established and delimited to be applied in the filters of collected articles. This adjustment allowed for a precise and efficient search of the available literature. As a result, a total of 91 articles were identified in PubMed, 109 from ScienceDirect, 30 from SpringerLink, and 93 from IEEE Explore based on the advanced search equations for each of the databases and search engines of scientific articles. Subsequently, the results were grouped, and duplicate results were eliminated to ensure the relevance of the selected studies. In addition, selection criteria were defined to include academic works consistent with the search terms to facilitate the classification and evaluation of the results, ensuring that only the most relevant and high-quality studies are considered in the systematic review. Articles that do not specifically focus on the identification of anxiety using AI techniques were discarded, as well as those that focused solely on depression without addressing anxiety. Studies that do not apply Machine Learning and Deep Learning algorithms and those that do not provide empirical data or quantitative results were excluded; publications that lack free and/or full access were also excluded. These criteria allowed for a more precise and focused selection of studies relevant to the objectives of the review.

Table 5 presents a set of exclusion criteria for a systematic review of studies related to artificial intelligence applied to anxiety disorders. These criteria, based on the acronym PICO (Patient/Population, Intervention, Comparison, Outcome/Result), serve as guides to select the studies that will be included in the analysis and exclude those that do not meet the established requirements.

**Table 5:** Exclusion and Inclusion criteria applied in all results of search equations.

Element	Exclusion criteria
P	CE1: Studies with populations that are not diagnosed with anxiety disorders, or that do not specify the anxiety disorder in question. Studies that have been conducted on animals or simulated samples.
I	CE2: The publication does not make contributions in Artificial Intelligence. Or qualitative approaches without evaluation.
C	CE3: Studies that do not include relevant information on Artificial Intelligence implementations or that are not evaluated or validated with metrics.
O	CE4: Studies that provide qualitative results without metrics.
Other criteria	CE5: Unauthorized access or through prior payment.
	CE6: Publications that are not in English or Spanish.
	CE7: Publications older than five years.
	CE8: Publications that are not strictly research articles.
Element	Inclusion criteria
P	CI1: Studies include individuals, groups, and/or populations with anxiety disorders.
I	CI2: Studies demonstrate the application of Artificial Intelligence algorithms and techniques for the diagnosis and/or identification of anxiety disorders. Additionally, studies that address NN, SVM, RF, and other DL and ML approaches are included.
C	CI3: RQ1 focuses on comparing the performance of various ML algorithms with each other, and RQ2 does so with ML techniques and traditional methods to assess accuracy.
O	CI4: Studies with results based on performance metrics such as accuracy, sensitivity, specificity, and area under the curve of algorithms used to identify anxiety disorders.
Other criteria	CI5: Studies published in peer-reviewed journals.
	CI6: The publication is not older than five years (2019–2024) to ensure the inclusion of recent and relevant research related to the diagnosis of anxiety disorders with AI.
	CI7: Publications in English and Spanish.

### 3.2 Eligibility criteria

For the analysis of the literature, studies that address the identification of anxiety using AI algorithms belonging to Machine Learning and Deep Learning were included. The selected studies provide empirical information and/or quantitative results and are published in peer-reviewed journals in the last five years, from 2019 to 2024, in four selected databases: IEEE Xplora, ScienceDirect, PubMed, and SpringerLink. The studies in English and Spanish encompass a greater diversity of research and full-access publications, allowing for a detailed analysis of their methodologies and findings. The decision to limit the search to articles in English and Spanish is based on the availability of resources and experience of the review team in these languages, recognizing that this may introduce a language bias and exclude relevant studies in other languages. To reduce language bias, we first worked on analyzing the summary/abstract to corroborate whether the information is relevant or not according to the established eligibility criteria in Table 5.

### 3.3 Data collection process

For the data collection process in this systematic review, the PRISMA Statement (Preferred Reporting Items for Systematic Reviews and Meta-Analyses) guidelines were followed (13). The data collection from the reports was carried out independently and based on the search terms specified in the methodology, which ensured objectivity and allowed for the identification of discrepancies. Productivity tools such as reference management software (14) and scientific article databases were used to identify and extract relevant information, facilitating the organization and analysis of large volumes of data. The collected data were recorded in an integrating table of references, and relevant information was extracted from each of the studies. To ensure uniformity in the collection of information, standardized templates and guides were developed that all reviewers followed rigorously. This structured and systematic approach allowed for a comprehensive and accurate data collection, in line with the best practices recommended by the PRISMA Statement.

### 3.4 Data items

Specific data related to the identification of anxiety disorders using Artificial Intelligence techniques were sought, focusing on key aspects such as AI algorithms, CNN, and other specific algorithms most frequently used in the selected studies. Validation data for the proposed methods were collected, including cross-validation, independent datasets, and training-test division, which allowed for the evaluation of the dimension and effectiveness of the implemented algorithms. The time points at which the results were measured were also recorded, such as the initial moment, during follow-up, and at the end of the study. This information allowed for the evaluation of the consistency and stability of the models over time. Data collection included a description of the methods used, specifying the sample size, the origin of the data (e.g., clinical data, self-reports, sensor data), and the type of data (structured or unstructured). This characterization of the data is crucial to understanding the context and applicability of the results. The inclusion criteria for the studies were based on the specificity of the approach to anxiety identification, the application of AI techniques, and the provision of quantitative results.

Likewise, data on any secondary analyzes performed in the studies, such as subgroup analyzes, adjustments for confounding variables, and sensitivity analyzes, were collected. This additional information allowed the analysis of factors that may influence the results. Therefore, studies with detailed descriptions and well-documented methodologies were prioritized, ensuring the reliability and relevance of the information gathered in this systematic review.

### 3.5 Study bias risk assessment

The reviewers independently and blindly evaluated the selected publications, ensuring that the work carried out was reliable. The Zotero reference management system (14) was used to organize the studies and facilitate the analysis of the sections of each selected study.

### 3.6 Synthesis methods

The process of this systematic review required a limited and defined method according to the eligibility criteria. The first step consisted of an exhaustive search in the IEEE, Elsevier, Scielo, and PubMed databases using query formulas with logical operators to guarantee a relevant range. Once all the studies were collected, duplicates were removed to ensure the integrity of the information gathered. The next step consisted of filtering the titles and content of the abstracts of all the selected studies, and those that met criteria CI1 (the publication directly addresses anxiety) and CI2 (the objectives related to the diagnosis and/or treatment of anxiety are evident in the abstract) were considered for the next stage.

The studies that passed the initial filter were read in their entirety. At this stage, it was necessary to apply CI3 (the authors’ contribution is significant to complement the systematic analysis) and CI4 (the use of Artificial Intelligence techniques for the diagnosis and/or treatment of anxiety is mentioned). To facilitate systematic comparison, detailed tables were created that included the key characteristics of each study, such as information on AI techniques, samples, results, study design, and validation methods, among others.

## 4 Results

### 4.1 Studies selected

**Figure 1:**
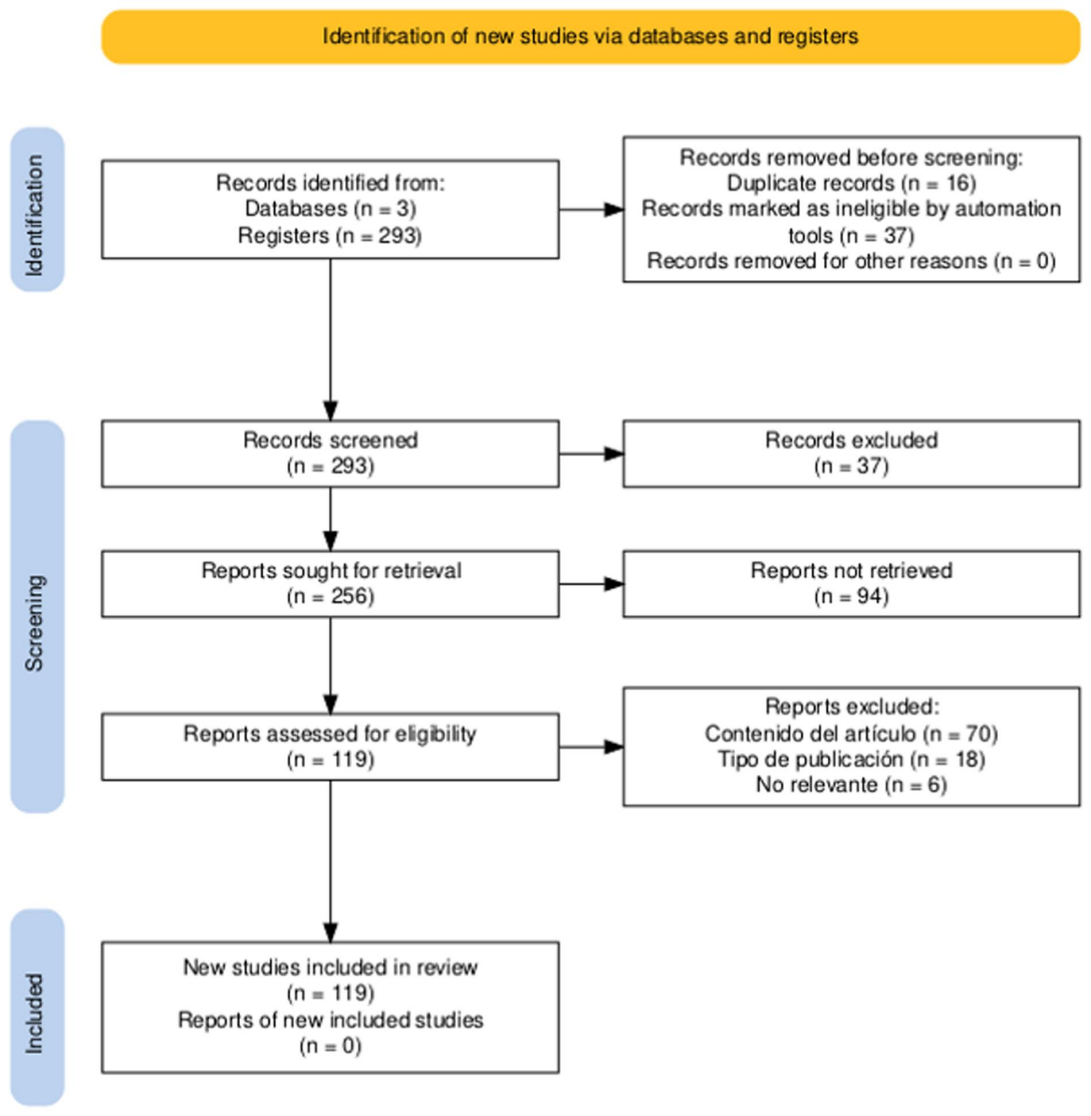
Result of the identification of research studies with PRISMA statement.

Figure 1 presents the PRISMA flow diagram (13) to illustrate the article selection process in a systematic review. It begins with the identification of studies through databases and records, which are subjected to an initial screening process to remove duplicates or those clearly not relevant. Next, the full text of the selected articles is obtained for a more detailed assessment of their eligibility, considering predefined criteria such as the content of the article or the type of publication. Finally, 119 studies included in the review are presented, thus demonstrating the transparency and rigor of the selection process carried out based on following inclusion criteria: studies include information specify of anxiety disorders (CI1), publications that demonstrate the application AI for the diagnosis anxiety disorders (CI2), information obtained of RQ1 and RQ2 contrast (CI3) and CI4–CI7 that contains common filters. The exclusion criteria has been applied in this Systematic Review after screening process. The following exclusion criteria where applied: studies with populations that are not diagnosed with anxiety disorders corresponding to CE1, publications does not make contributions in Artificial Intelligence (CE2), studies that do not include relevant information (CE3), studies that provide qualitative results without metrics (CE4) and CE5–CE8 that contains basic filters as last five years.

### 4.2 Individual study results

**Figure 2:**
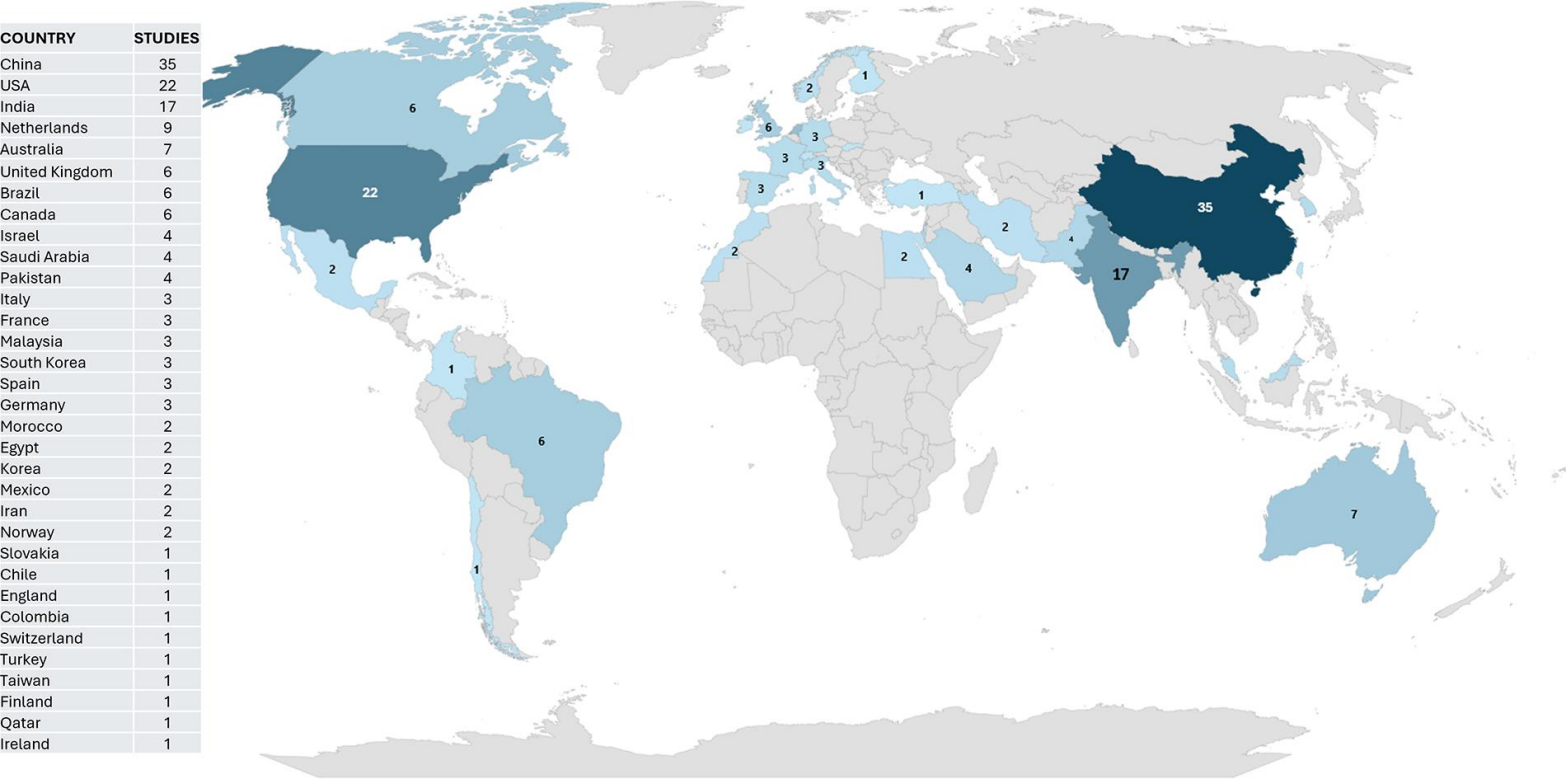
Distribution map of studies on anxiety disorders. Map created using Microsoft Excel.

Figure 2 reflects the number of studies, related to the search terms, published by country from January 2019 to September 2024. The concentration of studies on the application of artificial intelligence in mental health research is significant, particularly in China. This country, along with India and the United States, leads the vanguard in the development of innovative technological solutions for the diagnosis, treatment, and monitoring of mental disorders. The more intense coloration in these regions indicates a substantial investment in research and development in this emerging field.

The countries marked with a darker color indicate a greater number of published articles, among which China stands out with 35 studies, the United States with 22, and other countries with similar numbers. The trend of published studies indicates that in the northern hemisphere there is a constant research task within the study area and corresponds to the large number of technological news announced in various media. The following Artificial Intelligence techniques have been used in China: Natural Language Processing based on Machine Learning, DNN to identify complex characteristics in images and physiological signals within the diagnosis of anxiety. Similarly, CNN was used with the variant of identifying emotions in audios, others used predictive models of behavior in social networks. Very similar techniques were used in the United States, but with a focus on the analysis of emotions and anxiety disorders in social networks and clinical data in order to guarantee the reliability of the published results. In the rest of the countries, physiological signals, behavioral signals, and the fusion of characteristics applied to multimodal data were included.

Table 6 reflects how the predominant countries in the selected research area have contributed to improving the diagnosis of anxiety disorders. China and the United States stand out for using Machine Learning and Deep Learning in diverse populations made up of social media users (15–18) and patients with previous diagnoses of anxiety. Slovakia uses Deep Learning for the classification of emotions (arousal and valence) (19) with physiological signals, obtaining high accuracy results in the prediction of emotional states (12). Taiwan and Spain apply Deep Learning and Machine Learning techniques to clinical data (20), with the aim of improving the diagnostic accuracy of anxiety disorders by analyzing environmental factors and personal data.

**Table 6:** Predominant countries in the selected research area have contributed to improving the diagnosis of anxiety disorders.

Country	Artificial intelligence technique	Application
China	Deep learning, NLP for text analysis and emotion recognition	Emotion analysis, behavior prediction, clinical diagnosis
United States	Machine learning, neuroimaging analysis, NLP for social media	Social media analysis, neuroimaging for anxiety disorders
India	Hierarchical fusion of multimodal features, Deep Learning	Stress detection, affective signal analysis
United Kingdom	Machine learning, sentiment analysis in social media	Prediction of mental health impact by COVID-19
Netherlands	Machine learning applied to neuroimaging	Detection of neurocognitive abnormalities
Norway	Machine learning, sentiment analysis in social media	Emotional analysis in social media (COVID-19)
Slovakia	Deep learning for emotion classification (arousal and valence)	Classification of emotions from physiological signals
Taiwan	Deep learning in clinical and environment data	Diagnostic accuracy for anxiety and mental health
Spain	Machine learning, predictive models for anxiety analysis	Prediction of anxiety in adolescents
Australia	Deep learning, fusion of physiological and emotional features	Improvement in the detection of stress and emotions

### 4.3 Synthesis result

In the results of the application of PRISMA Statement (13), a total of 293 studies were identified from a database and three scientific article search engines. In the filtering process, 293 studies were selected to be reviewed more comprehensively, with a result of 37 articles excluded for not meeting the eligibility criteria defined in Table 9. Of the 256 studies that passed the selection phase, 119 studies were selected to be evaluated in depth to determine their eligibility. In this process, 94 studies were excluded due to various reasons: 70 for not providing relevant information, 18 for being an inappropriate type of publication, and 6 for being irrelevant. After the eligibility evaluation process, 119 studies were selected and included in this systematic review for analysis.

Of the 119 selected studies, it was identified that the majority use Artificial Intelligence techniques, including machine learning and deep learning, to identify anxiety disorders (21). These studies were based on self-reports (22), physiological data, and social media posts (70). Deep learning was used more in the studies examined due to the wide range of applications and the accuracy of the algorithms. As a result, it was found that these models were effective in detecting anxiety with great precision in contrast to traditional diagnostic tools that presented confidence biases in some cases. With the Systematic Review, some limitations were found in current research, including variability in the methods and data used, and possible bias in the training data.

Table 7 provides details of various studies that apply both Machine Learning and Deep Learning techniques in diverse populations for the identification of anxiety disorders, aligning with the research questions defined above. The filtered studies include from social media users (9, 23), to participants with anxiety disorders identified through neuroimaging (11) and patients with clinical data (24). This allows us to evaluate how different algorithms respond to heterogeneous populations, thus addressing research question RQ1. Interventions vary in the application of different Machine Learning and Deep Learning algorithms, focusing on the classification of anxiety disorders using social media (23, 25), while others apply methods such as the construction of emotional lexicons from large amounts of textual data (18), or the use of facial images and audio to assess mood states (26).

**Table 7:** Details of studies that apply both machine learning and deep learning techniques in diverse populations for the identification of anxiety disorders.

Country	P (population)	I (intervention)	C (comparator)	O (outcome)
China	Healthy individuals (20–24 years old)	Anxiety identification method based on deep features	Comparison with traditional methods	Superiority of deep features in anxiety identification
South Korea	English-speaking participants	Evaluation of model performance in autocorrect	Comparison with other autocorrect models	Accuracy, sensitivity, specificity of the model
Netherlands	Participants with anxiety (18–57 years old)	Neuroimaging study to detect morphological abnormalities	Control group without anxiety	Identification of neurocognitive abnormalities
Taiwan	Patients with clinical data	Analysis of diagnostic accuracy	Comparison with standard clinical metrics	Diagnostic accuracy of 72.4%
China	Participants with audio recordings and images	Evaluation of the Mood State Recognition System	Comparison with traditional systems	Effective recognition of mood state
Pakistan	Nurses	Detection of mental stress in nurses	Comparison with KNN-based approaches	Greater effectiveness in stress detection compared to KNN
India and Australia	Participants in different datasets	Stress detection through hierarchical fusion of features	No direct comparator	Improvement in stress detection
Saudi Arabia	Patients with clinical and pathological variables	Prediction of cardiovascular symptoms	Comparison with standard predictions	Accurate prediction of cardiovascular symptoms
Chile	Students enrolled in Computer Engineering	Prediction of technology adoption	Comparison with other adoption algorithms	Better predictions in technology adoption

Several studies in Table 7 include comparisons with traditional diagnostic methods or without a direct comparator, but also between different AI approaches. For example, in China one study identified an anxiety identification method based on deep features that demonstrated superiority over traditional methods. Similarly, in Taiwan, a study evaluated the diagnostic accuracy of an AI system using clinical data, obtaining a 72.4% accuracy and comparing it with standard clinical metrics. Chiu et al. (27), which clearly answers RQ2 by identifying the techniques that provide the greatest benefits in terms of diagnostic accuracy compared to traditional methods. Likewise, the results indicate significant improvements in the accuracy, sensitivity, and specificity of the proposed models, as observed in studies using Deep Learning (28, 29) and CNN (26). Another example is the classification of emotions using Deep Learning in Slovakia (2022), where high-precision results were obtained in predicting emotional states from physiological signals. These findings not only align with RQ2 by demonstrating which techniques optimize diagnostic tools, but also contribute to answering RQ1 by showing the high performance of these techniques in diverse populations and with varied data types.

Table 8 contains a summary of studies on Artificial Intelligence techniques used for the diagnosis of anxiety disorders, highlighting key metrics such as accuracy, sensitivity, and specificity. The use of Deep Learning demonstrates a more efficient performance in contrast to the handling of unstructured and multimodal data, as shown by studies conducted in China (2020) and Slovakia (2023). On the other hand, Machine Learning algorithms have also proven to be effective, especially in the analysis of brain images and texts, obtaining an accuracy of 90% in the detection of neurocognitive abnormalities in patients with anxiety and depression disorders. The use of Deep Learning for the classification of emotions in Slovakia (2023) was particularly effective, reaching accuracies of 87.88% in the classification of arousal and 85.61% in valence. This highlights how deep neural networks can process physiological signals to detect emotional states, which can be useful in the early identification of anxiety.

**Table 8:** Summary of studies on Artificial Intelligence techniques used for the diagnosis of anxiety disorders, highlighting key metrics such as accuracy, sensitivity, and specificity.

Year	Country	AI technique	Precision	Sensibility	Specificity
2020	China	Deep learning (DL)	87	85	83
2020	Norway	Machine learning (ML)—Sentiment analysis	82	N/A	N/A
2021	Netherlands	ML—Neuroimaging	90	88	85
2022	Taiwan	DL—Clinical data analysis	72.4	N/A	68.6
2023	Slovakia	DL—Emotion classification (Arousal y Valence)	87.88	N/A	85.61

Table 9 offers a global view of the various Artificial Intelligence (AI) techniques and algorithms used in research worldwide registered in the databases cited in this article between 2019 and 2024. Ranging from deep neural networks such as CNN and LSTM to machine learning algorithms such as Random Forest and SVM, the table illustrates the diversity of approaches used in AI. The combined use of CNN-LSTM-CNN in the United Kingdom, Deep Belief Networks (DBN) together with Soft-max Regression and the Limited-memory Broyden–Fletcher–Goldfarb–Shanno algorithm in Pakistan, and the implementation of the MACBETH method in Brazil (2019) are highlighted.

**Table 9:** Artificial Intelligence (AI) techniques and algorithms used in research worldwide registered in the databases cited in this article between 2019 and 2024.

Year	Country	Algorithms	Dataset	Data type	Details
2018	United Kingdom (30)	Deep learning (DL) techniques: convolutional neural network (CNN) and long short-term memory (LSTM) in a sandwich model (CNN-LSTM-CNN)	Natural-spontaneous affective dataset collected for this purpose, consisting of 862 videos of students with Asperger Syndrome (AS) and 545 videos for Typical Development (TD) students.	Video data (facial expressions, head movements, eye gaze, and occlusions like hand over face/head)	This model extracts natural affective states (confidence, uncertainty, engagement, anxiety, and boredom) of students with and without Asperger syndrome in a computer-based learning environment using a webcam, without requiring sensors or physiological instrumentation.
2019	Pakistan (23)	Co-training (semi-supervised learning) incorporating random forest (RF), support vector machine (SVM), and Naïve Bayes (NB)	Posts and comments from Reddit (subreddits: r/Depression, r/Anxiety, r/ADHD, r/Bipolar)	Text (posts are labeled, comments are unlabeled)	The study proposes a co-training-based methodology to classify mental illnesses (anxiety, depression, bipolar, ADHD) using social media posts from Reddit.
2019	Brazil (31)	Hybrid model combining a specialist (expert) system (based on production rules and probabilities using AI) with a multicriteria decision analysis method (MACBETH)	Not a traditional dataset, but rather a set of “control events” (symptoms and causes) for various psychological disorders, informed by the DSM-5 and expert psychiatric and psychological reports.	Qualitative and comparative analysis of events and criteria.	The psychological disorders addressed include schizophrenia spectrum disorders, bipolar disorder, depressive disorders, anxiety disorders, obsessive-compulsive disorder, trauma-related disorders and stressors.
2020	Norway (25)	LSTM models for sentiment polarity and emotion detection. The proposed multi-layer LSTM assessment model used FastText, GloVe, and GloVe Twitter pre-trained embeddings.	Custom collected trending hashtag data (February 2020) and publicly available Kaggle dataset (March-April 2020) for COVID-19 related tweets.	Text (Tweets)	This study aimed to analyze cross-cultural reactions to the COVID-19 pandemic using sentiment and emotion detection on tweets from six neighboring countries across three continents (Pakistan, India, Norway, Sweden, USA, Canada).
2020	United Arab Emirates, USA (32)	Convolutional neural networks (CNNs), specifically AlexNet, with transfer learning. Feature fusion using Support Vector Machine (SVM) for final classification. Comparisons were made with K-Star, K-Nearest Neighbor (kNN), Random Forest, and Random Tree classifiers.	Optical Coherence Tomography (OCT) images from 52 subjects (26 with Non-Proliferative Diabetic Retinopathy (NPDR) and 26 normal), collected at the Kentucky Lions Eye Center at University of Louisville. Images are 1024x1024 pixels, 8-bit grayscale. Transfer learning used a subset of the ImageNet database (1.2 million images).	Medical images (OCT scans)	The system involves preprocessing (retina layer segmentation, fovea detection, patch extraction and alignment), CNN-based feature extraction, and SVM-based classification.
2021	South Korea (33)	Logistic regression models, specifically a second-order polynomial logistic regression model for the drawing experiment and a first-order (linear) logistic regression model for the proofreading experiment.	Two experimental datasets: 1. Drawing software (Google AutoDraw) experiment: 18 participants. 2. English proofreading software experiment: 19 native English speakers (18 after data exclusion).	Physiological signals (Electrodermal Activity - EDA) and task success/failure data.	Participants used two types of AI software (drawing and English proofreading), and their EDA was measured as a stress indicator. Stress levels were classified as low or high.
2022	India (34)	Deep neural network (DNN) with a joint modality auto-encoder (JMAE) for joint modality feature learning, and a Convolutional Recurrent Neural Network with Squeeze-Excitation modules (CRNN-SE) as the classifier. Different cost functions (MSE, Cosine similarity, KL divergence) were investigated for the auto-encoder	Four benchmark datasets: ASCERTAIN (58 subjects), CLAS (62 subjects), MAUS (22 subjects), and WAUC (48 participants). The first 42, 43, 18, and 36 subject samples from ASCERTAIN, CLAS, MAUS, and WAUC datasets, respectively, were used for training, with the remainder used for testing.	Physiological signals: Electrodermal Activity (EDA) and Electrocardiogram (ECG).	Joint features were learned using an auto-encoder, and then used to train a CRNN-SE classifier for differentiating stressed and unstressed subjects.
2022	Mexico (35)	The study utilizes results from the Global Burden of Disease (GBD) 2021 study, which itself uses modeling and methods to correct for underreporting and account for mortality and morbidity.	Data for Mexico from the Global Burden of Disease (GBD) 2021 study, including estimates from 1990 to 2021. Mental disorders were grouped according to DSM-IV-TR and ICD-10 diagnostic criteria.	Epidemiological data: prevalence, incidence, years lived with disability (YLDs), years of healthy life lost (DALYs), disaggregated by sex, age, and federal entity.	It estimated 18.1 million people with a mental disorder in 2021, a 15.4% increase from 2019. Depressive and anxiety disorders significantly increased between 2019 and 2021, possibly related to COVID-19, confinement, and grief.
2023	United Kingdom (9)	Hybrid deep learning model: Recurrent Neural Network (in the form of Long Short-Term Memory or LSTM) and Convolutional Neural Network (CNN), termed LSTM-CNN. For comparison, Generalized LSTM, Logistic Regression (LR), Linear Support Vector (LSV), Naive Bayes (NB), and SVM models were used	Twitter data (tweets): Sentiment 140 dataset (1.6 million tweets, labeled as positive or negative) and Depressive Tweets Processed dataset (2345 depressive tweets, manually verified).	Text (Twitter)	A novel hybrid LSTM-CNN model is proposed, capable of identifying depressive tweets with an accuracy of 99.42%.
2023	China (26)	Combination of grounded theory (qualitative research) and semi-automatic methods (Word2Vec for word expansion, manual filtering). For emotion recognition, a lexicon-based and rule-based approach was used.	7,535 Weibo texts were used for coding and theoretical model development (Study 1). For word expansion (Study 2), a corpus of 1.01 million Weibo texts was collected.	Text (Weibo posts)	It first used a bottom-up approach with grounded theory to derive a theoretical model for emotions expressed on Weibo, leading to eight core emotion categories: joy, expectation, love, anger, anxiety, disgust, sadness, and surprise. Second, a lexicon of 2,964 words was built by manually selecting seed words, expanding them using a Word2Vec model, and filtering.
2023	Mexico, Canada (3)	Machine learning: SVM (with linear and RBF kernels), KNN, Decision Tree Classifier, Random Forest Classifier, and Multi-layer Perceptron Classifier.	27 participants (19–44 years old) playing a First Person Shooter (FPS) Virtual Reality (VR) video game with three difficulty levels and rest stages.	Physiological signals: ECG, EDA, EMG.	For classification between the three difficulty levels, an 83.1% accuracy was obtained with a KNN model using EDA and ECG features. When all features from ECG, EDA, and EMG signals were used, an accuracy of 99% was obtained for differentiating between the three difficulty levels and a resting stage.
2023	India, Australia (18)	Feature ensemble based Bayesian neural network (FE-BNN). It exploits Markov Chain Monte Carlo approximation for sampling. Compared with traditional classifiers (logistic regression, SVM, LDA, Naïve Bayes), neural networks (MLP, DNN, BNN, DNN (Dropout)), and ensemble methods (Random Forest, AdaBoost, Gradient Tree Boosting). Feature selection was performed with Lasso.	Three disorder-specific anxiety datasets collected by the online tool YODA (Youth Online Diagnostic Assessment): Separation Anxiety Disorder (39 cases, 30 controls, 69 × 19 questions), Generalized Anxiety Disorder (76 cases, 95 controls, 171 × 32 questions), and Social Anxiety Disorder (58 cases, 74 controls, 132 × 28 questions). Participants were children and adolescents aged 6 to 16 (mean age 9.34 years), with parent-reported responses.	Online questionnaire data (binary responses or severity/frequency scales).	The method achieved AUCs of 0.8683, 0.8769, and 0.9091 for separation anxiety disorder, generalized anxiety disorder, and social anxiety disorder predictions, respectively.

Table 10 describes a selection of Deep Learning algorithms, a subcategory of machine learning that focuses on the use of artificial neural networks with multiple layers to analyze data and extract complex patterns. The table presents algorithms such as LSTM, used for sequence analysis; Q-Learning, a reinforcement learning algorithm; and CNN-LSTM-CNN, an architecture that combines convolutional and recurrent neural networks. It also includes machine learning algorithms such as SVM, Naive Bayes, and Decision Tree, which, although not exclusive to Deep Learning, are often used in conjunction with deep neural networks to improve their performance.

**Table 10:** Algorithms identified for results search equations.

Algorithm	Description
LSTM (long short-term memory) (26, 36–44)	A recurrent neural network specifically designed to learn patterns in data sequences such as text or time series.
Q-learning (45)	A reinforcement learning algorithm that allows an agent to learn to make optimal decisions in an environment through interaction and feedback.
CNN-LSTM-CNN (36, 38, 45–55)	An architecture that combines two CNN networks with an intermediate LSTM layer, used for tasks such as action recognition in videos.
SVM (support vector machine) (56–68),	A supervised learning algorithm that is used for classification and regression. It seeks a hyperplane that maximizes the separation between classes.
Naïve bayes (8, 17, 27, 59, 60, 69–81)	A probabilistic classification algorithm based on Bayes’ theorem, which assumes that the features are conditionally independent given the class.
Decision tree (31, 48, 53, 82–92)	A tree model that represents decisions and their possible consequences in the form of a hierarchical structure.

## 5 Discussion

Anxiety disorders stand out because they directly affect the mental health of the individual who suffers from them, so early and accurate identification is crucial to generate effective treatment and improve prognosis and then channel them to the corresponding areas and begin appropriate treatment. This systematic review aimed to provide a comprehensive analysis of Artificial Intelligence (AI) techniques and methodologies applied to anxiety disorder detection, focusing on their accuracy and research scope, in response to our core research questions: RQ1 (What Artificial Intelligence techniques show the best performance in identifying anxiety disorders in diverse populations?) and RQ2 (What methodologies and approaches have been adopted to train and validate Artificial Intelligence techniques in identifying anxiety disorders?).

Regarding RQ1, the findings indicate a prevalent application of both Machine Learning (ML) and Deep Learning (DL) techniques across the analyzed studies, as highlighted in Tables 8, 9. Deep Learning, in particular, demonstrated a more efficient performance in handling unstructured and multimodal data, and was used more frequently due to its broad range of applications and high algorithm accuracy. For instance, studies in China (2020) and Slovakia (2023) using DL showed high accuracies, such as 87% for anxiety detection and 87.88% for arousal classification from physiological signals, respectively. ML algorithms also proved effective, especially in brain image and text analysis, achieving accuracies of 90% in detecting neurocognitive abnormalities. These AI-driven methods generally exhibited higher accuracy compared to traditional anxiety disorder detection tests, offering valuable insights for improved diagnostic tools.

In response to RQ2, a diversity of methodologies and approaches were adopted for training and validating these AI techniques. Studies utilized varied data sources, including self-reports, physiological data (e.g., heart rate, galvanic skin response, neuroimaging like fMRI, EEG), voice data, text data (from social media, questionnaires), and behavioral data. Validation methods typically involved cross-validation, independent datasets, and training-test divisions to evaluate algorithm effectiveness and consistency over time. Comparisons with traditional diagnostic methods were frequently observed, demonstrating the superiority of AI techniques in terms of diagnostic accuracy, sensitivity, and specificity. Specific algorithms identified included LSTM, Q-Learning, CNN-LSTM-CNN, SVM, Naive Bayes, Decision Tree, Random Forest, and Deep Neural Networks, reflecting a broad spectrum of AI applications.

However, despite these promising advances, it is crucial to recognize that scientific research in this area is still in an early stage of development, at least in Mexico, and faces several significant challenges that limit its broader implementation and generalization. These challenges not only reside in the application of AI, but also in the characteristics of the data and the methodologies employed in the existing studies.

It should be noted that a large part of the filtered studies focused on samples that were often small and homogeneous, raising significant questions about the generalizability of the results to more diverse populations in real clinical practice. This intrinsic limitation restricts the applicability of the developed artificial intelligence models, as their performance could degrade considerably when facing the broad demographic (e.g., age, gender), cultural (e.g., ethnicity, cultural origin), and clinical diversity characteristic of the global population. Another important challenge lies in the inherent variability of training datasets and the imperant need to ensure their representativeness. Data collection, especially from subjective sources such as self-reports or from physiological and social media data, can introduce subtle, but significant, biases into the models. These biases can originate from the specific method of data collection, the participants’ interpretation of symptoms, or even from imbalances between data classes that do not accurately reflect the actual prevalence of anxiety disorders. For example, the dependence on social media data, as observed in several studies, often lacks clinical validation and may reflect a specific and unrepresentative subset of the population. Similarly, the comorbidity of anxiety with other mental health disorders (lines 74–75) and the subjectivity of symptoms (lines 40–41) pose inherent challenges for obtaining entirely “clean” and unbiased contextual training data. While the aspiration is for thorough, precise, and well-defined work to guarantee the reliability of results from their origin, the heterogeneity in data collection and processing methodologies across the various studies analyzed also severely limits the comparability of results and hinders the identification of the most universally effective models or clear “best practices.” This lack of methodological standardization, coupled with the particularities of each dataset, makes comparative performance evaluation complex and the replicability of findings in different environments a persistent obstacle.

Furthermore, it was observed that some studies focus on areas that, while relevant to AI, could be considered overexploited in the context of anxiety detection, such as Natural Language Processing in comments from social networks like Twitter and Reddit, without offering substantial progress in overcoming the aforementioned limitations. The lack of controlled experiments that allow the methodology to be applied in contexts different from those proposed in the published studies also presents a significant barrier to the transfer of technology to the clinical setting.

## 6 Conclusions

Artificial Intelligence is experiencing potential growth in the development of systems for the early and accurate diagnosis of anxiety disorders, which will allow for faster and more effective treatment. However, it should be noted that research in this area is still ongoing and poses significant challenges. Therefore, future research should address these challenges using larger and more diverse samples, and standardizing methods and data. Future research should be guided by an experimental approach applied to reality in various contexts, conducting more extensive and rigorous studies to verify the effectiveness of AI models in diverse settings and populations. In general, data should be more diverse in terms of age, gender, ethnicity, and cultural origin; establish standards and procedures for collecting, analyzing, and interpreting data from AI studies on anxiety. In conclusion, AI offers a promising perspective for improving the detection and treatment of anxiety. However, it is necessary to address existing challenges and conduct further research to validate AI models, ensure their fairness, and consider the ethical implications of their use. Collaboration between researchers, mental health professionals, and AI experts will be crucial to make the most of the potential of this technology and improve the lives of people suffering from anxiety disorders.

## Data Availability

The original contributions presented in the study are included in the article/Supplementary Material, further inquiries can be directed to the corresponding author.

## References

[1] Ramírez-Ortiz J Castro-Quintero D Lerma-Córdoba C Yela-Ceballos F Escobar-Córdoba F. Data from: Consecuencias de la pandemia covid 19 en la salud mental asociadas al aislamiento social. (2020). 10.1590/SciELOPreprints.303

[2] Delgado EC Lara MF Arias RM. Generalidades sobre el trastorno de ansiedad. (2021).

[3] Medina-Mora ME Orozco R Rafful C Cordero M Bishai J Ferrari A, et al. Los trastornos mentales en méxico 1990–2021. Resultados del estudio. Glob Burden Dis. (2023) 159:12335. 10.24875/GMM.23000376 38386882

[4] Passot Y. Miedo a la muerte y ansiedad en el contexto COVID-19. Rev Cient Arbitr Fund MenteClara. (2022) 7:1–9. 10.32351/rca.v7.308

[5] Fernández-Martínez E Liébana-Presa C Martínez-Soriano M López-Alonso A. Miedo a la muerte y su relación con la inteligencia emocional en estudiantes del último curso de enfermería. Med Paliat. (2019) 26:205–10. 10.20986/medpal.2019.1063/2019

[6] Valdez-Santiago R Marín-Mendoza E Torres-Falcón M. Análisis comparativo del marco legal en salud mental y suicidio en México. Salud Publica Mex. (2021) 63:554–64. 10.21149/12310 35078289

[7] Hernández Hernández VA Marrufo RM Esparza Del Villar OA Robles Ramírez AJ. Validación de las escalas de beck (ansiedad y depresión) en población fronteriza (norte de México) durante la pandemia por COVID-19. Sapienza Int J Interdiscipl Stud. (2022) 3:155–68. 10.51798/sijis.v3i5.452

[8] Almadhor A Abbas S Sampedro GA Alsubai S Ojo S Hejaili AA, et al. Multi-class adaptive active learning for predicting student anxiety. IEEE Access. (2024) 12:58097–105. 10.1109/ACCESS.2024.3391418

[9] Banna MHA Ghosh T Nahian MJA Kaiser MS Mahmud M Taher KA, et al. A hybrid deep learning model to predict the impact of COVID-19 on mental health from social media big data. IEEE Access. (2023) 11:77009–22. 10.1109/ACCESS.2023.3293857

[10] Ansari L Ji S Chen Q Cambria E. Ensemble hybrid learning methods for automated depression detection. IEEE Trans Comput Soc Syst. (2023) 10:211–9. 10.1109/TCSS.2022.3154442

[11] Van Tol M Van Der Wee N Veltman D. Fifteen years of NESDA neuroimaging: an overview of results related to clinical profile and bio-social risk factors of major depressive disorder and common anxiety disorders. J Affect Disord. (2021) 289:31–45. 10.1016/j.jad.2021.04.009 33933910

[12] Schiavenato M Chu F. PICO: what it is and what it is not. Nurse Educ Pract. (2021) 56:103194. 10.1016/j.nepr.2021.103194 34534728

[13] Page MJ McKenzie JE Bossuyt PM Boutron I Hoffmann TC Mulrow CD, et al. The PRISMA 2020 statement: an updated guideline for reporting systematic reviews. BMJ. (2021) 372:n71. 10.1136/bmj.n71 33782057 PMC8005924

[14] Kaur S Dhindsa KS. Comparative study of citation and reference management tools: Mendeley, Zotero and ReadCube. In: *2016 International Conference on ICT in Business Industry & Government (ICTBIG)*. IEEE (2016). p. 1–5.

[15] Ahuja K. Emotion AI in healthcare: application, challenges, and future directions. In: Garg M Koundal D, editors. Emotional AI and Human-AI Interactions in Social Networking. London: Academic Press (2024). p. 131–46.

[16] Al-Abyadh MHA Hoang VT. Emotion AI: cognitive behavioral therapy for teens having some mental health disorders. In: Garg M, Koundal D, editors. *Emotional AI and Human-AI Interactions in Social Networking*. London: Academic Press (2024). p. 169–89.

[17] Ali M Baqir A Husnain Raza Sherazi H Hussain A Hassan Alshehri A Ali Imran M. Machine learning based psychotic behaviors prediction from facebook status updates. Comput Mater Contin. (2022) 72:2411–27. 10.32604/cmc.2022.024704

[18] Xu L Li L Jiang Z Sun Z Wen X Shi J, et al. A novel emotion lexicon for Chinese emotional expression analysis on weibo: using grounded theory and semi-automatic methods. IEEE Access. (2021) 9:92757–68. 10.1109/ACCESS.2020.3009292

[19] Sadeghi M McDonald AD Sasangohar F Kaiser MS. Posttraumatic stress disorder hyperarousal event detection using smartwatch physiological and activity data. PLoS One. (2022) 17:e0267749. 10.1371/journal.pone.0267749 35584096 PMC9116643

[20] Sharma A Verbeke WJMI Li Z. Understanding importance of clinical biomarkers for diagnosis of anxiety disorders using machine learning models. PLoS One. (2021) 16:e0251365. 10.1371/journal.pone.0251365 33970950 PMC8109802

[21] Reyes Marrero R De Portugal Fernández Del Rivero E. Trastornos de ansiedad. Medicine. (2019) 12:4911–7. 10.1016/j.med.2019.07.001

[22] Liu X-L Chang L-S. Deciphering the genetic links between psychological stress, autophagy, and dermatological health: Insights from bioinformatics, single-cell analysis, and machine learning in psoriasis and anxiety disorders. Int J Mol Sci. (2024) 25:5387. 10.3390/ijms25105387 38791423 PMC11121097

[23] Tariq S Akhtar N Afzal H Khalid S Mufti MR Hussain S, et al. A novel co-training-based approach for the classification of mental illnesses using social media posts. IEEE Access. (2019) 7:166165–72. 10.1109/ACCESS.2019.2953087

[24] Wu C-T Wang S-M Su Y-E Hsieh T-T Chen P-C Cheng Y-C, et al. A precision health service for chronic diseases: development and cohort study using wearable device, machine learning, and deep learning. IEEE J Transl Eng Health Med. (2022) 10:1–14. 10.1109/JTEHM.2022.3207825 PMC9529197 36199984

[25] Imran AS Daudpota SM Kastrati Z Batra R. Cross-cultural polarity and emotion detection using sentiment analysis and deep learning on COVID-19 related tweets. IEEE Access. (2020) 8:181074–90. 10.1109/ACCESS.2020.3027350 34812358 PMC8545282

[26] Xiong J Yin H Pan M. Application of image classification based on improved LSTM in internet reading therapy platform. IEEE Access. (2024) 12:1660–71. 10.1109/ACCESS.2023.3347346

[27] Chiu YM Sirois C Simard M Gagnon M-E Talbot D. Traditional methods hold their ground against machine learning in predicting potentially inappropriate medication use in older adults. Value Health. (2024) 27:1393–9. 10.1016/j.jval.2024.06.005 38977181

[28] Deng A Yang Y Li Y Huang M Li L Lu Y, et al. Using machine learning algorithm to predict the risk of post-traumatic stress disorder among firefighters in Changsha. Zhong Nan Da Xue Xue Bao Yi Xue Ban. (2023) 48:84–91. 10.11817/j.issn.1672-7347.2023.220067 36935181 PMC10930560

[29] Wu J Yang J Yuan Z Zhang J Zhang Z Qin T, et al. Functional connectome gradient predicts clinical symptoms of chronic insomnia disorder. Prog Neuro-Psychopharmacol Biol Psychiatry. (2024) 135:111120. 10.1016/j.pnpbp.2024.111120 39154930

[30] Dawood A Turner S Perepa P. Affective computational model to extract natural affective states of students with asperger syndrome (AS) in computer-based learning environment. IEEE Access. (2018) 6:67026–34. 10.1109/ACCESS.2018.2879619

[31] Nunes LC Pinheiro PR Dantas Pinheiro MC Simao Filho M Comin Nunes RE Dantas Pinheiro PGC. Automatic detection and diagnosis of neurologic diseases. IEEE Access. (2019) 7:29924–41. 10.1109/ACCESS.2019.2899216

[32] Tian Y Fan R Ding X Zhang X Gan T. Predicting rumor retweeting behavior of social media users in public emergencies. IEEE Access. (2020) 8:87121–32. 10.1109/ACCESS.2020.2989180

[33] Bitkina OV Kim J Park J Park J Kim HK. User stress in artificial intelligence: modeling in case of system failure. IEEE Access. (2021) 9:137430–43. 10.1109/ACCESS.2021.3117120

[34] Radhika K Subramanian R Oruganti VRM. Joint modality features in frequency domain for stress detection. IEEE Access. (2022) 10:57201–11. 10.1109/ACCESS.2022.3178409

[35] Orozco-Mora CE Oceguera-Cuevas D Fuentes-Aguilar RQ Hernandez-Melgarejo G. Stress level estimation based on physiological signals for virtual reality applications. IEEE Access. (2022) 10:68755–67. 10.1109/ACCESS.2022.3186318

[36] Al-Ezzi A Yahya N Kamel N Faye I Alsaih K Gunaseli E. Severity assessment of social anxiety disorder using deep learning models on brain effective connectivity. IEEE Access. (2021) 9:86899–913. 10.1109/ACCESS.2021.3089358

[37] Al-Abyadh MHA Hoang VT. Chapter nine—emotion AI: cognitive behavioral therapy for teens having some mental health disorders. In: Garg M, Koundal D, editors. *Emotional AI and Human-AI Interactions in Social Networking*. London: Academic Press (2024). p. 169–89.

[38] Du G Tan Q Li C Wang X Teng S Liu PX. A noncontact emotion recognition method based on complexion and heart rate. IEEE Trans Instrum Meas. (2022) 71:1–14. 10.1109/TIM.2022.3194858

[39] Kamakshi K Rengaraj A. Early detection of stress and anxiety based seizures in position data augmented EEG signal using hybrid deep learning algorithms. IEEE Access. (2024) 12:35351–65. 10.1109/ACCESS.2024.3365192

[40] Khan NS Ghani MS Anjum G. ADAM-sense: anxiety-displaying activities recognition by motion sensors. Pervasive Mob Comput. (2021) 78:101485. 10.1016/j.pmcj.2021.101485

[41] Kumar A. Using cognition to resolve duplicacy issues in socially connected healthcare for smart cities. Comput Commun. (2020) 152:272–81. 10.1016/j.comcom.2020.01.041

[42] Mou L Zhao Y Zhou C Nakisa B Rastgoo MN Ma L, et al. Driver emotion recognition with a hybrid attentional multimodal fusion framework. IEEE Trans Affect Comput. (2023) 14:2970–81. 10.1109/TAFFC.2023.3250460

[43] Tian X Zhu L Zhang M Wang S Lu Y Xu X, et al. Social anxiety prediction based on ERP features: a deep learning approach. J Affect Disord. (2024) 367:545–53. 10.1016/j.jad.2024.09.006 39236887

[44] Tsai C-H Christian M Kuo Y-Y Lu CC Lai F Huang W-L. Sleep, physical activity and panic attacks: a two-year prospective cohort study using smartwatches, deep learning and an explainable artificial intelligence model. Sleep Med. (2024) 114:55–63. 10.1016/j.sleep.2023.12.013 38154150

[45] Moosavi SKR Zafar MH Sanfilippo F Akhter MN Hadi SF. Early mental stress detection using q-learning embedded starling murmuration optimiser-based deep learning model. IEEE Access. (2023) 11:116860–78. 10.1109/ACCESS.2023.3326129

[46] Zogan H Razzak I Jameel S Xu G. Hierarchical convolutional attention network for depression detection on social media and its impact during pandemic. IEEE J Biomed Health Inform. (2024) 28:1815–23. 10.1109/JBHI.2023.3243249 37022816

[47] Mortensen JA Mollov ME Chatterjee A Ghose D Li FY. Multi-class stress detection through heart rate variability: a deep neural network based study. IEEE Access. (2023) 11:57470–80. 10.1109/ACCESS.2023.3274478

[48] Aina J Akinniyi O Rahman MM Odero-Marah V Khalifa F. A hybrid learning-architecture for mental disorder detection using emotion recognition. IEEE Access. (2024) 12:91410–25. 10.1109/ACCESS.2024.3421376 39054996 PMC11270886

[49] Zhu J Zhang Z Guo Z Li Z. Sentiment classification of anxiety-related texts in social media via fuzing linguistic and semantic features. IEEE Trans Comput Soc Syst. (2024) 11:6819–29. 10.1109/TCSS.2024.3410391

[50] Mokatren LS Ansari R Cetin AE Leow AD Ajilore OA Klumpp H, et al. EEG classification by factoring in sensor spatial configuration. IEEE Access. (2021) 9:19053–65. 10.1109/ACCESS.2021.3054670

[51] López M-J Arias CP Romeu J Jofre-Roca L. Supervised machine learning-assisted driving stress monitoring MIMO radar system. IEEE Sens J. (2023) 23:28899–911. 10.1109/JSEN.2023.3326880

[52] Villa-Pérez ME Trejo LA Moin MB Stroulia E. Extracting mental health indicators from English and Spanish social media: a machine learning approach. IEEE Access. (2023) 11:128135–52. 10.1109/ACCESS.2023.3332289

[53] Sriram Kumar P Govarthan PK Aleem Shaik Gadda A Ganapathy N Fredo Agastinose Ronickom J. Deep learning-based automated emotion recognition using multimodal physiological signals and time-frequency methods. IEEE Trans Instrum Meas. (2024) 73:1–12. 10.1109/TIM.2024.3420349

[54] Kuttala R Subramanian R Oruganti VRM. Multimodal hierarchical CNN feature fusion for stress detection. IEEE Access. (2023) 11:6867–78. 10.1109/ACCESS.2023.3237545

[55] Meng X Zhang J. Anxiety recognition of college students using a takagi-sugeno-kang fuzzy system modeling method and deep features. IEEE Access. (2020) 8:159897–905. 10.1109/ACCESS.2020.3021092

[56] Oduntan A Oyebode O Beltran AH Fowles J Steeves D Orji R. “I let depression and anxiety drown me…”: identifying factors associated with resilience based on journaling using machine learning and thematic analysis. IEEE J Biomed Health Inform. (2022) 26:3397–408. 10.1109/JBHI.2022.3149862 35139031

[57] Bu X Hu X Zhang L Li B Zhou M Lu L, et al. Investigating the predictive value of different resting-state functional MRI parameters in obsessive-compulsive disorder. Transl Psychiatry. (2019) 9:17. 10.1038/s41398-018-0362-9 30655506 PMC6336781

[58] Ghazal TM Al Hamadi H Umar Nasir M Atta-Ur-Rahman MG Zubair M Khan MA, et al. Supervised machine learning empowered multifactorial genetic inheritance disorder prediction. Comput Intell Neurosci. (2022) 2022:1051388. 10.1155/2022/1051388 35685134 PMC9173933

[59] Huma S Sohail MK Akhtar N Muhammad D Afzal H Mufti MR, et al. Analyzing COVID-2019 impact on mental health through social media forum. CMES. (2021) 67:3737–48. 10.32604/cmc.2021.014398

[60] Zhu L Spachos P Ng PC Yu Y Wang Y Plataniotis K, et al. Stress detection through wrist-based electrodermal activity monitoring and machine learning. IEEE J Biomed Health Inform. (2023) 27:2155–65. 10.1109/JBHI.2023.3239305 37022004

[61] Mohamed ES Naqishbandi TA Bukhari SAC Rauf I Sawrikar V Hussain A. A hybrid mental health prediction model using support vector machine, multilayer perceptron, and random forest algorithms. Healthc Anal. (2023) 3:100185. 10.1016/j.health.2023.100185

[62] Na K-S Cho S-E Cho S-J. Machine learning-based discrimination of panic disorder from other anxiety disorders. J Affect Disord. (2021) 278:1–4. 10.1016/j.jad.2020.09.027 32942220

[63] Ximenes de Brito R Rolim Fernandes CA Martins Moreira RM Oliveira EN. Prediction model for common mental disorder and depression in users of psychoactive drugs. IEEE Latin Am Trans. (2023) 21:399–407. 10.1109/TLA.2023.10068843

[64] Schultebraucks K Qian M Abu-Amara D Dean K Laska E Siegel C, et al. Pre-deployment risk factors for PTSD in active-duty personnel deployed to Afghanistan: a machine-learning approach for analyzing multivariate predictors. Mol Psychiatry. (2021) 26:5011–22. 10.1038/s41380-020-0789-2 32488126 PMC8589682

[65] Tabares Tabares M Vélez Álvarez C Bernal Salcedo J Murillo Rendón S. Anxiety in young people: analysis from a machine learning model. Acta Psychol. (2024) 248:104410. 10.1016/j.actpsy.2024.104410 39032273

[66] Yang X Hu X Tang W Li B Yang Y Gong Q, et al. Multivariate classification of drug-naive obsessive-compulsive disorder patients and healthy controls by applying an SVM to resting-state functional MRI data. BMC Psychiatry. (2019) 19:210. 10.1186/s12888-019-2184-6 31277632 PMC6612132

[67] Zhang J Richardson JD Dunkley BT. Classifying post-traumatic stress disorder using the magnetoencephalographic connectome and machine learning. Sci Rep. (2020) 10:5937. 10.1038/s41598-020-62713-5 32246035 PMC7125168

[68] Zhao C Hu S He T Yuan L Yang X Wang J, et al. Deep learning-based identification of common complication features of surgical incisions. Sichuan Da Xue Xue Bao Yi Xue Ban. (2023) 54:923–9. 10.12182/20230960303 37866947 PMC10579068

[69] Al-Ezzi A Al-Shargabi AA Al-Shargie F Zahary AT. Complexity analysis of EEG in patients with social anxiety disorder using fuzzy entropy and machine learning techniques. IEEE Access. (2022) 10:39926–38. 10.1109/ACCESS.2022.3165199

[70] Baqir A Ali M Jaffar S Sherazi HHR Lee M Bashir AK, et al. Identifying COVID-19 survivors living with post-traumatic stress disorder through machine learning on twitter. Sci Rep. (2024) 14:18902. 10.1038/s41598-024-69687-8 39143145 PMC11325037

[71] Bone C Simmonds-Buckley M Thwaites R Sandford D Merzhvynska M Rubel J, et al. Dynamic prediction of psychological treatment outcomes: development and validation of a prediction model using routinely collected symptom data. Lancet Digit Health. (2021) 3:e231–40. 10.1016/S2589-7500(21)00018-2 33766287

[72] Wickramasuriya DS Crofford LJ Widge AS Faghih RT. Hybrid decoders for marked point process observations and external influences. IEEE Trans Biomed Eng. (2023) 70:343–53. 10.1109/TBME.2022.3191243 35839187

[73] Delamain H Buckman JEJ O’Driscoll C Suh JW Stott J Singh S, et al. Predicting post-treatment symptom severity for adults receiving psychological therapy in routine care for generalised anxiety disorder: a machine learning approach. Psychiatry Res. (2024) 336:115910. 10.1016/j.psychres.2024.115910 38608539

[74] Oğur NB Çeken C Oğur YS Yuvaci HU Yazici AB Yazici E. Development of an artificial intelligence-supported hybrid data management platform for monitoring depression and anxiety symptoms in the perinatal period: pilot-scale study. IEEE Access. (2023) 11:31456–66. 10.1109/ACCESS.2023.3262467

[75] Faruqui SHA Alaeddini A Wang J Jaramillo CA Pugh MJ. A functional model for structure learning and parameter estimation in continuous time bayesian network: an application in identifying patterns of multiple chronic conditions. IEEE Access. (2021) 9:148076–89. 10.1109/ACCESS.2021.3122912 35371895 PMC8975131

[76] Shikha D Sethia D Indu S. Optimization of wearable biosensor data for stress classification using machine learning and explainable AI. IEEE Access. (2024) 12:169310. 10.1109/ACCESS.2024.3463742

[77] Shobhika Kumar P Chandra S. Prediction and comparison of psychological health during COVID-19 among Indian population and Rajyoga meditators using machine learning algorithms. Int Conf Mach Learn Data Eng. (2023) 218:697–705. 10.1016/j.procs.2023.01.050 PMC9886327 36743799

[78] Smith DL Held P. Moving toward precision PTSD treatment: predicting veterans’ intensive PTSD treatment response using continuously updating machine learning models. Psychol Med. (2023) 53:5500–9. 10.1017/S0033291722002689 36259132 PMC10482723

[79] van Eeden WA Luo C van Hemert AM Carlier IV Penninx BW Wardenaar KJ, et al. Predicting the 9-year course of mood and anxiety disorders with automated machine learning: A comparison between auto-sklearn, Naïve Bayes classifier, and traditional logistic regression. Psychiatry Res. (2021) 299:113823. 10.1016/j.psychres.2021.113823 33667949

[80] Xiong H Berkovsky S Romano M Sharan RV Liu S Coiera E, et al. Prediction of anxiety disorders using a feature ensemble based bayesian neural network. J Biomed Inform. (2021) 123:103921. 10.1016/j.jbi.2021.103921 34628061

[81] Zarate D Ball M Prokofieva M Kostakos V Stavropoulos V. Identifying self-disclosed anxiety on Twitter: a natural language processing approach. Psychiatry Res. (2023) 330:115579. 10.1016/j.psychres.2023.115579 37956589

[82] Pampouchidou A Simos PG Marias K Meriaudeau F Yang F Pediaditis M, et al. Automatic assessment of depression based on visual cues: a systematic review. IEEE Trans Affect Comput. (2019) 10:445–70. 10.1109/TAFFC.2017.2724035

[83] Deshpande G Masood J Huynh N Denney TS Dretsch MN. Interpretable deep learning for neuroimaging-based diagnostic classification. IEEE Access. (2024) 12:55474–90. 10.1109/ACCESS.2024.3388911

[84] Bhattacharya J Gupta A Dretsch MN Denney TS Deshpande G. A reliable clinical decision support system for post traumatic stress disorder using functional magnetic resonance imaging data. IEEE Trans Artif Intell. (2024) 5:5605–15. 10.1109/TAI.2024.3411596

[85] Woodward K Kanjo E Brown DJ McGinnity TM Inkster B Macintyre DJ, et al. Beyond mobile apps: a survey of technologies for mental well-being. IEEE Trans Affect Comput. (2022) 13:1216–35. 10.1109/TAFFC.2020.3015018

[86] Tong L Liu Z Jiang Z Zhou F Chen L Lyu J, et al. Cost-sensitive boosting pruning trees for depression detection on twitter. IEEE Trans Affect Comput. (2023) 14:1898–911. 10.1109/TAFFC.2022.3145634

[87] Zare MA Boostani R Mohammadi M Kouchaki S. A dopamine based adaptive emotional neural network. IEEE Access. (2022) 10:109460–75. 10.1109/ACCESS.2022.3212403

[88] Huang M Zhang X Chen X Mai Y Wu X Zhao J, et al. Joint-channel-connectivity-based feature selection and classification on fNIRS for stress detection in decision-making. IEEE Trans Neural Syst Rehabil Eng. (2022) 30:1858–69. 10.1109/TNSRE.2022.3188560 35788456

[89] Bhavani T VamseeKrishna P Chakraborty C Dwivedi P. Stress classification and vital signs forecasting for IoT-health monitoring. IEEE/ACM Trans Comput Biol Bioinform. (2024) 21:652–9. 10.1109/TCBB.2022.3196151 35921342

[90] Guo T Zhao W Alrashoud M Tolba A Firmin S Xia F. Multimodal educational data fusion for students’ mental health detection. IEEE Access. (2022) 10:70370–82. 10.1109/ACCESS.2022.3187502

[91] Borba de Souza V Campos Nobre J Becker K. DAC stacking: a deep learning ensemble to classify anxiety, depression, and their comorbidity from reddit texts. IEEE J Biomed Health Inform. (2022) 26:3303–11. 10.1109/JBHI.2022.3151589 35230959

[92] Elnakieb YA Ali MT Soliman A Mahmoud AH Shalaby AM Alghamdi NS, et al. Computer aided autism diagnosis using diffusion tensor imaging. IEEE Access. (2020) 8:191298–308. 10.1109/ACCESS.2020.3032066

